# A new 12-gene diagnostic biomarker signature of melanoma revealed by integrated microarray analysis

**DOI:** 10.7717/peerj.49

**Published:** 2013-03-05

**Authors:** Wanting Liu, Yonghong Peng, Desmond J. Tobin

**Affiliations:** 1 Department of Computing, University of Bradford, Great Britain; 2 Centre of Skin Sciences, University of Bradford, Great Britain

**Keywords:** Gene biomarker, Microarray, Bioinformatics, Genome, Nevi, Skin, Melanocytes, Melanoma, Metastasis, Immunochemistry

## Abstract

Genome-wide microarray technology has facilitated the systematic discovery of diagnostic biomarkers of cancers and other pathologies. However, meta-analyses of published arrays often uncover significant inconsistencies that hinder advances in clinical practice. Here we present an integrated microarray analysis framework, based on a genome-wide relative significance (GWRS) and genome-wide global significance (GWGS) model. When applied to five microarray datasets on melanoma published between 2000 and 2011, this method revealed a new signature of 200 genes. When these were linked to so-called ‘melanoma driver’ genes involved in MAPK, Ca^2+^, and WNT signaling pathways we were able to produce a new 12-gene diagnostic biomarker signature for melanoma (i.e., *EGFR, FGFR2, FGFR3, IL8, PTPRF, TNC, CXCL13, COL11A1, CHP2, SHC4, PPP2R2C*, and *WNT4*). We have begun to experimentally validate a subset of these genes involved in MAPK signaling at the protein level, including *CXCL13, COL11A1, PTPRF* and *SHC4* and found these to be over-expressed in metastatic and primary melanoma cells *in vitro* and *in situ* compared to melanocytes cultured from healthy skin epidermis and normal healthy human skin. While SHC4 has been reported previously to be associated to melanoma, this is the first time *CXCL13, COL11A1*, and *PTPRF* have been associated with melanoma on experimental validation. Our computational evaluation indicates that this 12-gene biomarker signature achieves excellent diagnostic power in distinguishing metastatic melanoma from normal skin and benign nevus. Further experimental validation of the role of these 12 genes in a new signaling network may provide new insights into the underlying biological mechanisms driving the progression of melanoma.

## Introduction

Melanoma is a cancer involving the transformation and uncontrolled growth of melanocytes (Miller & Mihm, 2006) and can originate in skin, mucosa, uvea, and leptomeninges (Eigentler & Garbe, 2006). Since the mid-1960s the reported incidence of melanoma has increased every year by up to 8% (Lens, 2008). Malignant melanoma metastasizes quickly and only 14% of patients with metastatic disease can expect to live for 5 years (Miller & Mihm, 2006). While some new therapies are coming on stream (e.g., ipilimumab) (Postow et al., 2012), the cure rate largely depends on early detection and tumor removal by surgery. Metastatic potential is mainly related to tumor thickness (Rigel & Carucci, 2000), and a greater than 90% cure rate is possible if the tumor is less than 1 mm thick when removed (Gremel et al., 2009).

A robust genetic marker signature should greatly advance both the diagnosis and targeted treatment of melanoma in clinical practice. To that end, microarray technology has been used as an advanced high-throughput strategy for the discovery of diagnostic gene signatures of human diseases at the genome-wide scale. The genome-wide discovery of such a signature would provide important insights into the underlying biological mechanisms driving melanomagenesis. A significant amount of microarray data has been produced and deposited in publically-available data repositories recently, including Gene Expression Omnibus (GEO) (Barrett et al., 2011) and ArrayExpress Archive (Parkinson et al., 2011). These repositories allow scientists to advance the discovery of diagnostic and prognostic gene signatures by means of data integration and bioinformatics analysis. Lukk et al. (2010) constructed a global map of human gene expression by integrating microarray data from 5,372 human samples representing 369 different cell and tissue types, disease states and cell lines. While microarray technology has also been applied to comparative analyses of different stages in melanoma development and have identified various gene signatures (Hoek, 2007), there is poor congruence between gene signatures generated by different microarray-based melanoma studies (John et al., 2008; Bittner et al., 2000; Tímár, Gyorffy & Rásó, 2010). Unsurprisingly therefore, microarray-based melanoma gene biomarkers have had poor translation to clinical practise, and melanoma diagnosis is still based on clinical and histopathological features of the tumor (Schramm et al., 2011).

Meta-analysis approaches have been used to seek out and reveal often latent data complexity and connectivity, and so have the potential to increase the robustness of data interpretation (Ramasamy et al., 2008; Hong & Breitling, 2008; Cochran & Conn, 2008). Choi and co-workers (Choi et al., 2003) have demonstrated that meta-analysis can positively influence statistical significance by amending the false negative rate of individual studies. Using this approach, Rhodes and colleagues successfully identified 50 over-expressed and 103 under-expressed genes in an enhanced signature of prostate cancer (Rhodes et al., 2002). Similarly, Parmigiani and co-workers built a cross-study comparison for lung cancer (Parmigiani et al., 2004), while Park and Stegall revealed the true involvement of cytokine genes of human kidney disease by combining their own microarray data with other public sources (Park & Stegall, 2007).

Two very recent reviews and meta-analyses of melanoma microarray studies (Tímár, Gyorffy & Rásó, 2010; Schramm et al., 2011) revealed some strikingly contradictory results. Tímár et al. compared signatures derived from four microarray datasets of human melanoma tissue, but found very little overlap between the signatures, both within and between these studies (Tímár, Gyorffy & Rásó, 2010). They attributed much of this lack of congruence to sample heterogeneity. By adding 5 additional studies, Schramm and colleagues however demonstrated some significant over-represented functions among the melanoma gene signatures (Schramm et al., 2011); especially those related to the immune response. A ‘leave-one-out’ cross validation with a low average error rate (28%) across all validation expression data was achieved for the gene signature of Mann, Pupo & Campain (2013).

To identify a more robust gene biomarker signature for melanoma we propose a new model that measures the genome-wide relative significance (GWRS) and genome-wide global significance (GWGS) of gene expression. This new model enables the integrative analysis of microarray datasets produced by different platforms and protocols. We examined microarray-based melanoma studies published between 2000 and 2011 and retrieved five microarray datasets that study differential gene expression between normal skin and/or benign nevi and metastatic melanoma (Hoek et al., 2004; Smith, Hoek & Becker, 2005; Riker et al., 2008; Scatolini et al., 2010; Rose et al., 2011). The integrated analysis of these five microarray datasets identified a robust biomarker signature of 12 genes for melanoma, which includes six previously-unreported genes. Our integrated investigation combines a computational approach with experimental validation.

## Methods

### Microarray datasets

This study examines the differential expression of genes between normal skin and/or benign nevi, and metastatic melanoma using a meta-analysis approach. The experimental protocol of this study is shown in Fig. 5 and commenced with the identification of 16 microarray studies on metastatic melanoma published 2000 to 2011. Microarray data included in these studies are shown in Table S5. In the current study, we focused our attention on the differential gene expression between normal skin and/or benign nevi and metastatic melanoma. On this basis four microarray datasets were extracted (GEO access number: GSE7553, GSE4587, GSE4579, and GSE12391). An additional GSE22301 dataset was extracted from Rose et al. (2011), but while this study did not provide a gene signature of metastatic melanoma (and so was not included in the meta-analysis of 16 studies) it did include 14 samples of metastatic melanoma data and so was included in our integrative analysis. Thus, a total of five microarray datasets of normal and/or benign nevi and metastatic melanoma were used in this study (Table S6).

### Genome-wide relative significance (GWRS) and Genome-wide global significance (GWGS) for integrated analysis of cross-laboratory microarray data

A relatively simple method of integrative meta-analysis was proposed by Rhodes et al. in 2002 that combines independent microarray studies based on the *p*-value of each individual gene:

\begin{eqnarray*} \displaystyle {s}_{p}=-\sum _{i=1}^{n}2\log ({p}_{i})&&\displaystyle \end{eqnarray*}

where *p*_*i*_, *i* = 1–*n*, is the *p*-value of a gene in the *i*-th independent study. However, this method has at least two significant limitations: (1) many microarray studies are based on a small number of samples, for which the *p*-value can therefore be problematic and (2) the large variation in *p*-values across different studies leads to the data with smallest *p*-value determining the outcome of *S*_*p*_.

We propose a new approach based on measuring the genome-wide relative significance (GWRS) and genome-wide global significance (GWGS) of expressed genes. We measure the GWRS of a gene using its ranking position (Jurman et al., 2008) on a genome-wide scale (*r* value) based on a differential expression measure, which can be the fold change, *t*-test *p*-value, SAM (Significance Analysis of Microarray data) *p*-value etc. Most existing meta analysis methods focus on the top-*k* genes (e.g. Jurman et al., 2008), while our method counts the ranking of genome-wide genes in total. Compared to the model of Rhodes and co-workers the proposed approach possess two important enhancements: (1) it can apply multiple different methods for measuring the degree of differential expression of a gene (e.g. fold change, *t*-test, Anova or SAM *p*-values) and (2) it uses a ranking *r* value instead of the test statistic (i.e., fold change, or *p*-value) to avoid the influence of high variation test statistics.

### Data preparation

Pre-processing of microarray data is performed by extracting the expression value for each individual gene from the associated probe-sets. When a probe-set is mapped to multiple genes, e.g. ‘209994_s_at’ associated to two genes ‘ABCB1 / ABCB4’ in GSE4570, both genes are given the expression of the ‘209994_s_at’ probe-set.

For a gene appearing in multiple probe-sets, the most significant differential expressed probe-sets are assigned to this gene. We tested the results of using mean-, median-, and maxim-based methods to deal with the situations were multiple probe-sets are associated to a gene. We observed that the maxim-based method was able to retrieve the most significant probe-set of a gene, and would reflect our aim of extracting the most competitive genes across multiple studies. By contrast, use of a mean- or median-based probe-set value of a gene would drag the expression level down, and may introduce bias in follow-up analysis. As a result, a list of unique genes (G) from the datasets was retrieved. The number of datasets was denoted by *n*, while the number of unique genes across *n* datasets was denoted by *m*, i.e. *m* = |G|. The value ‘NA’ was applied in cases where a gene is absent from an individual study. We removed a gene from G where NA is bigger than δ (δ = 2 in this study), i.e. a gene was removed if it is absent for more than two of five datasets. This resulted in *m* = 24,097 and *n* = 5.

### Measuring the GWRS of genes in each single microarray database

For each gene in the list of unique genes (G), we measured the degree of differential expression that can be measured by fold-change, *t*-test (*p*-value), SAM or other statistical test. However, fold-change is used in the current study, as our computational evaluation indicated that this produces more reliable results, probably due to the limited number of samples in some of the datasets. For each gene in G, we assigned a rank number (in descending order starting from 1 to *m*) according to their corresponding degree of differential expression i.e. a gene with a high degree of differential expression was ranked more highly and so with a smaller ranking number. An *m*∗*n* matrix (*R*) was thus created in which *r*_*ij*_ is the ranking number of the *i*-th gene in the *j*-th dataset. We measure the GWRS of the *i*-th gene in the *j*-th dataset by:

\begin{eqnarray*} \displaystyle {s}_{i j}=-2 \hspace{0.167em} \log \left(\frac{{r}_{i j}}{m}\right)&&\displaystyle \end{eqnarray*}

where *r*_*ij*_, *i* = 1–*m*, *j* = 1–*n*, is the rank number of the *i*-th gene in the *j*-th study. The range of GWRS value (*s*_*ij*_) is between 0 and −2log(1/*m*). For a gene with ‘NA’ value the *s*_*ij*_ is set to be ‘NA’.

### Measuring the GWGS of a gene across multiple microarray datasets

We estimated the GWGS (${s}_{i}^{r}$) of a gene based on its corresponding GWRS across *n* datasets, by

\begin{eqnarray*} \displaystyle {s}_{i}^{r}=\sum _{j=1}^{n}{\omega }_{j}{s}_{i j}&&\displaystyle \end{eqnarray*}

where ω_*j*_ represents the relative weight of the *j*-th dataset, and ${\mathop{\sum }\nolimits }_{j=1}^{n}{\omega }_{j}=1$. The value of weight (ω_*j*_) can be assigned based on the data quality of the *j*-th datasets (e.g. the level of data noise. The value of ω_*j*_ can also be used to reflect the differential importance of biopsy versus cell line samples that biological scientists may wish to take into account. In this study, we treated all the dataset equally, thus the weight of each datasets was set equally to be 1/*n* for *j* = 1–*n*. We also selected only the top 200 genes from the full gene list for further analysis (i.e. selected genes with the greatest *s*^*r*^ value) by empirical evaluation of the classification performance (accuracy ratio). This was determined using the ‘wrapper-feature selection’ after multiple rounds of gene addition (ranging from 20 genes up to 500 genes) in order to distinguish melanoma from normal skin/benign nevus. We observed that using more than 200 genes yielded no improvement in classification ratio values, and so we consider 200 genes as an optimal gene set with the smallest number of genes that still can achieve a similar level of classification performance.

### Pathway analysis

We performed a pathway analysis to assess functional relevance of the new 200 gene signature based on the DAVID database (Hosack et al., 2003). DAVID provides a useful tool to analyze large gene lists, including via gene ontology and pathway analysis. We applied our top 200 genes to this database in order to detect potentially over-represented KEGG pathways. Before inputting into the DAVID database, we extracted the corresponding probe-sets of the 200 genes for the corresponding microarray platforms of each dataset. In comparison with the gene signature in the original 16 studies, we also extracted their associated probe-sets. We retrieved 31 pathways from the KEGG database where 12 genes (i.e., *EGFR, FGFR2, FGFR3, IL8, PTPRF, TNC, CXCL13, COL11A1, CHP2, SHC4, PPP2R2C,* and *WNT4*) in this 200-gene signature were found to closely interact with the 4 melanoma driver genes (see Results section).

### Immunocytochemistry (ICC)

Primary epidermal melanocyte (EM) (female 44y), moderately pigmented human melanoma cells (FM55), and highly pigmented human melanoma cells (FM94) (melanoma cells were a gift of Dr Janis Ancans, University of Latvia) were cultured as previously described (Gledhill et al., 2010). The cells were fixed in ice-cold methanol (Sigma, Poole, Dorset, UK) for 10 min before air drying and rehydration in PBS. The cells were blocked with 10% donkey serum (DS) for 1 h, washed with PBS before incubation with respective primary antibodies to four test antigens from this 12-gene signature. These included: COL11A1 (Abcam, ab64883), CXCL13 (R & D Systems, AF801), PTPRF (NeuroMab, 75-193), SHC4 (Proteintech, 12641-1-AP), which were incubated overnight at 4 °C followed by secondary antibody (1:300) for 1 h (donkey anti-goat (Invitrogen, A11055), donkey anti-mouse (Invitrogen, A21202), donkey anti-rabbit (Invitrogen, A21206), Alexa green). The slides were cover-slipped by VECTASHIELD mounting medium with DAPI and photographed using a Nikon Eclipse 80i fluorescence microscope and imaged with a Nikon Digital Sight DS-U1 camera. A full assessment of all 12 proteins in our melanoma signature is beyond the scope of the current study, but will be assessed in detail in a follow-up studies.

### Double immunohistochemistry (IHC)

Paraffin-embedded primary melanoma *in situ* (nose) and metastatic melanoma (lower leg) were deparaffinized and boiled in sodium citrate buffer (10 mM, 0.05% Tween 20, pH 6.0) for antigen retrieval. Acetone-fixed cryosections of normal human facial skin (Female 52 yrs) were used as control samples. All tissues were blocked with 10% donkey serum (DS) for 1 h, washed with PBS before 2 h incubation with NKi/beteb antibody raised against the melanocyte lineage-specific marker gp100 as a positive pigment cell control (Monosan; Mon7006-1) (1:15) followed by each of the 4 test antibodies at room temperature.

### Data Access

The microarray data used in this study were retrieved from Gene Expression Omnibus (GEO) with the following access numbers: GSE4570, GSE4587, GSE7553, GSE12391, and GSE22301. The 16 signatures of melanoma reported in the literature between 2000 and 2011 were extracted from the associated publication and is presented in Table S2.

## Results

### Gene signatures of melanoma (2000 to 2011) share few common genes

A meta-analysis conducted on gene signatures of metastatic melanoma reported in 16 independent microarray-based studies (ranging from 5 to 589 genes/study) from 2000 to 2011, showed remarkably few shared genes (Table 1, and Supplementary Information Table S1).

**Table 1:** Pairwise comparisons of 16 independent studies of melanoma and their associated distribution of common genes.^*^

	Alonso et al., 2007 (243)	Bogunovic et al., 2009 (209)	Haqq et al., 2005 (19)	Hoek et al., 2004 (589)	Jaeger et al., 2007 (308)	Jeffs et al., 2009 (96)	John et al., 2008 (21)	Kabbarah et al., 2010 (30)	Kashani-sabet et al., 2009 (5)	Koh et al., 2009 (14)	Mandruzzato et al., 2006 (71)	Okamoto et al., 2005 (20)	Riker et al., 2008(65)	Scatolini et al., 2010 (455)	Smith, Hoek & Becker, 2005 (94 of 100)	Winneperninckx et al., 2006 (235)
Alonso et al., 2007 (243)		2	1	12	4	2	0	1	1	0	0	0	1	5	1	7
Bogunovic et al., 2009 (209)			1	9	3	2	0	2	0	0	6	0	2	1	0	6
Haqq et al., 2005 (19)				7	1	0	0	0	0	0	0	0	0	1	1	1
Hoek et al., 2004 (589)					34	19	0	7	1	2	5	2	8	17	6	4
Jaeger et al., 2007 (308)						3	0	1	2	0	1	1	27	84	9	14
Jeffs et al., 2009 (96)							0	0	0	0	2	2	0	0	2	1
John et al., 2008 (21)								0	0	0	0	0	0	0	0	1
Kabbarah et al., 2010 (30)									0	0	0	0	2	1	0	3
Kashani-sabet et al., 2009 (5)										0	0	0	1	0	1	0
Koh et al., 2009 (14)											0	0	0	0	1	0
Mandruzzato et al., 2006 (71)												0	0	3	0	3
Okamoto et al., 2005 (20)													0	0	0	0
Riker et al., 2008 (65)														16	7	4
Scatolini et al., 2010 (455)															4	14
Smith, Hoek & Becker, 2005 (94 of 100)																1
Winneperninckx et al., 2006 (235)																

**Notes.**

* The numbers in brackets are the number of genes in the orginal study signatures. The 4 microarray datasets used in the current study are highlighted with underline.

There were 84 genes common to two of the signatures (Scatolini et al., 2010; Jaeger et al., 2007), while 14 common genes appeared in three studies (Scatolini et al., 2010; Jaeger et al., 2007; Riker et al., 2008). Strikingly, while there were only 2 genes (KRT15, RORA) in common in four of the 16 studies (Scatolini et al., 2010; Jaeger et al., 2007; Riker et al., 2008; Smith, Hoek & Becker, 2005), we have recognized four genes in our 200 gene set (i.e. KRT15, MAGEA6, RORA and SULF1) that appeared in 4 different studies of the 16. No gene was common in five or more independent studies (Table S2). This finding suggested that there may be some fundamental issues with either the manner in which these microarray studies were designed, or with the meta-analyses conducted. On this basis we set about designing a new more robust model for meta-analysis.

### Integrated analysis of cross-laboratory microarray data reveal a new melanoma gene signature

We applied our new approach to integratively analyze five independent microarray studies (Hoek et al., 2004; Smith, Hoek & Becker, 2005; Riker et al., 2008; Scatolini et al., 2010; Rose et al., 2011) (see Methods). The genome wide ‘global significance’ or GWGS of a gene (i.e., across all five datasets) was measured by the GWGS (*s*^*r*^) as defined above (see Methods). A gene with a large *s*^*r*^ value is considered to be significant across multiple independent studies (i.e., globally significant). The 200 genes with largest *s*^*r*^ value were selected as the starting point for our new proposed gene signature of melanoma, as listed in Table 2 and Table S3. This set of 200 signature genes was empirically determined, based on the classification accuracy ratio after various rounds of gene additions (using the ‘wrapper feature selection’ approach) in order to distinguish melanoma from normal skin cells and/or benign nevus. As the classification accuracy ratio was improved very little by adding more than 200 genes, we applied this gene set as the smallest number of genes to retain the optimal classification accuracy performance.

**Table 2:** The 200 genes with largest *s*^*r*^ values that were selected as the proposed gene signature of melanoma.

No.	Genes	No.	Genes	No.	Genes	No.	Genes
1	DCD	51	GAGE7	101	DKFZP434B061	151	AQP3
2	MAGEA3	52	DGAT2	102	PPP1R14C	152	C1orf116
3	MAGEA2	53	FGFR3	103	AKR1C3	153	RGS4
4	MAGEA2B	54	MICALCL	104	C19orf33	154	GRHL3
5	CSAG3	55	KRT15	105	FGFR2	155	GPR115
6	CSAG2	56	CTAG2	106	IGL@	156	SERPINA3
7	GAGE12F	57	ANK3	107	SERPINB5	157	LAD1
8	GAGE12G	58	HMGA2	108	CYP3A5	158	FLI37464
9	GAGE12I	59	MYOZ2	109	LEP	159	HLA-DRB4
10	GAGE2A	60	AADACL2	110	CHST6	160	TMEM79
11	GAGE2B	61	SCGB2A2	111	TF	161	ZNF750
12	GAGE2C	62	ISG20	112	MIA	162	IGHV4-31
13	GAGE2E	63	DST	113	HLA-DQB1	163	TP63
14	GAGE4	64	IL13RA2	114	GPR87	164	LOC124220
15	CTAG1B	65	APOC2	115	RHBDL2	165	RASGRF1
16	KRT77	66	TNC	116	SGPP2	166	KRT5
17	THRSP	67	FMN2	117	SCARA5	167	LAMB4
18	CTAG1A	68	SHC4	118	SAA1	168	SCML4
19	GAGE5	69	FSTL5	119	RNASE2	169	CYP4B1
20	GAGE6	70	PTPRF	120	SLAMF7	170	HLA-DRB3
21	MAGEA12	71	KRTAP19-1	121	SAA2	171	NEBL
22	MAGEA6	72	CXCL13	122	PPP2R2C	172	IGSF9
23	XAGE1A	73	GAGE1	123	GBP5	173	KLK11
24	XAGE1B	74	EYA1	124	AKR1C1	174	CHP2
25	XAGE1C	75	HLA-DRB2	125	ENTHD1	175	MAGEA10
26	XAGE1D	76	LOC100133484	126	EPHA3	176	CYP26B1
27	XAGE1E	77	LOC100133661	127	KRT6B	177	EREG
28	PRAME	78	LOC100133811	128	CCDC3	178	DLX1
29	C4orf7	79	LOC730415	129	BTBD16	179	LOC285986
30	GAGE12B	80	ZNF749	130	ANKRD35	180	TRIM7
31	GAGE12C	81	KRT14	131	HLA-DQA1	181	GAD1
32	GAGE12D	82	IGFL2	132	C10orf116	182	LOR
33	GAGE12E	83	SCEL	133	JUP	183	EXPH5
34	GAGE12H	84	GAGE3	134	IGFBP5	184	TMEM154
35	GAGE12J	85	GATA3	135	KRT25	185	LASS3
36	GAGE2D	86	DSP	136	SULF1	186	HLA-DRB5
37	GAGE8	87	WNT4	137	TKTL1	187	LOC100126583
38	WFDC5	88	TACSTD2	138	IL1F7	188	CYP4F8
39	IL8	89	CAPNS2	139	C6orf218	189	SDC1
40	COL17A1	90	MAL2	140	HEY1	190	SCGB1D2
41	FOXQ1	91	DGAT2L3	141	MGST1	191	RORA
42	ZIC1	92	PIP	142	ABCA13	192	SH3RF2
43	ELMOD1	93	AKR1C2	143	RAPGEFL1	193	LGALS7
44	ELOVL3	94	IGF2	144	TFPI2	194	MMP1
45	SERPINA12	95	MPP7	145	TRIM29	195	MAGEC1
46	DSC3	96	IGHG1	146	ALDH1A3	196	FRMD5
47	MAGEA1	97	NMU	147	ATP6V1C2	197	SERPINB7
48	DMKN	98	EGFR	148	COL11A1	198	FGF13
49	INS-IGF2	99	APOC4	149	RSPO1	199	LOC645323
50	C1orf172	100	MGP	150	PLA1A	200	COL9A3

### Validation of a new 200-gene signature based on experimental studies reported in the literature

**Figure 1:**
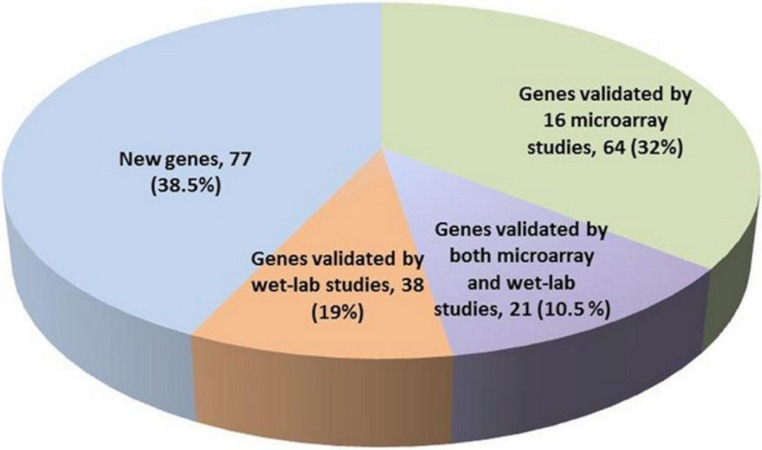
Validation of the proposed 200-gene signature. The 200 signature gene set is taken from the full list of genes associated with melanoma and was selected for further analysis based on their classification accuracy ratio (i.e. genes with the greatest *s*^*r*^ value).

The 200 genes found to have genome-wide global significance in our study were compared with the gene signatures identified in previously-published reports (Table S5). Our new 200-gene signature was first validated by (i) comparing it with 16 signatures proposed in the referred to set of microarray studies (Table S1), (ii) checking if any experimental validation of these genes was published in the literature (PubMed, last access: 16 April 2012). This analysis revealed that (a) 85 genes in our 200-gene signature were reported in at least one of the 16 microarray studies, and (b) 21 genes of the 200-gene signature were reported in both microarray studies and wet-lab experimental studies (Table S4, labeled yellow background). We also found that 38 genes of this 200-gene signature were not reported in any of the 16 reference studies, but had in fact been previously validated in independent wet-lab studies (Table S4 and discussion section). Importantly, our new gene signature reported an additional subset of 77 genes that were not previously reported anywhere in the literature in association with melanoma (Fig. 1). The ranking positions of these 77 genes shows that 39% appear in the top 100 and 34% in bottom 50 (see Table S7). These genes may represent ‘novel genes’ as they were not previously identified in published microarray studies. We further investigated the characteristics of the 85 genes reported in at least 1 of the 16 reference microarray studies (Table S3). Forty-four were reported in ≥ 2 studies, while 17 genes have been reported in ≥ 3 of the 16 studies (Table S3). KRT15, MAGEA6, RORA and SULF1 were the most frequently reported genes appearing in 4 of the 16 studies. Thus, using our method, we are able to pick up 4 of the 7 most frequently reported genes in the 16 studies by using just our top 200 genes (i.e., 30% less than the next best list of 308 genes in Jaeger et al. (2007)). In this way the methodology to select the top 200 genes in our study is more powerful than previously reported on the component 16 published signatures used for the source data (Table 1).

### Interaction of a new 200-gene signature with melanoma ‘driver’ genes informs a new signaling network in melanoma

We investigated the interaction between genes within our 200-gene signature with the four known melanoma ‘driver’ genes (i.e., *NRAS, BRAF, MITF* and *cKIT*). Of these driver genes, NRAS is mutated in 13–25% of melanoma cases (Goel et al., 2006; Schubbert, Shannon & Bollag, 2007), while *BRAF* (located downstream of *NRAS*), is mutated in up to 45% of malignant melanomas (Hocker & Tsao, 2007; Flaherty & McArthur, 2010). MITF, a master transcription factor in melanocyte function, cooperates when mutated with BRAF in melanomagenesis (Garraway et al., 2005; Taylor et al., 2011). Recent studies show that mutant *cKIT* can activate the Ras/Raf/Mek/Erk pathway and also activate *MITF* (Monsel et al., 2009; Phung et al., 2011). The four well-known melanoma driver genes did not appear on our list. This is due most likely to these four driver genes being associated with melanoma at the gene mutation level, rather than at the gene expression level.

**Figure 2:**
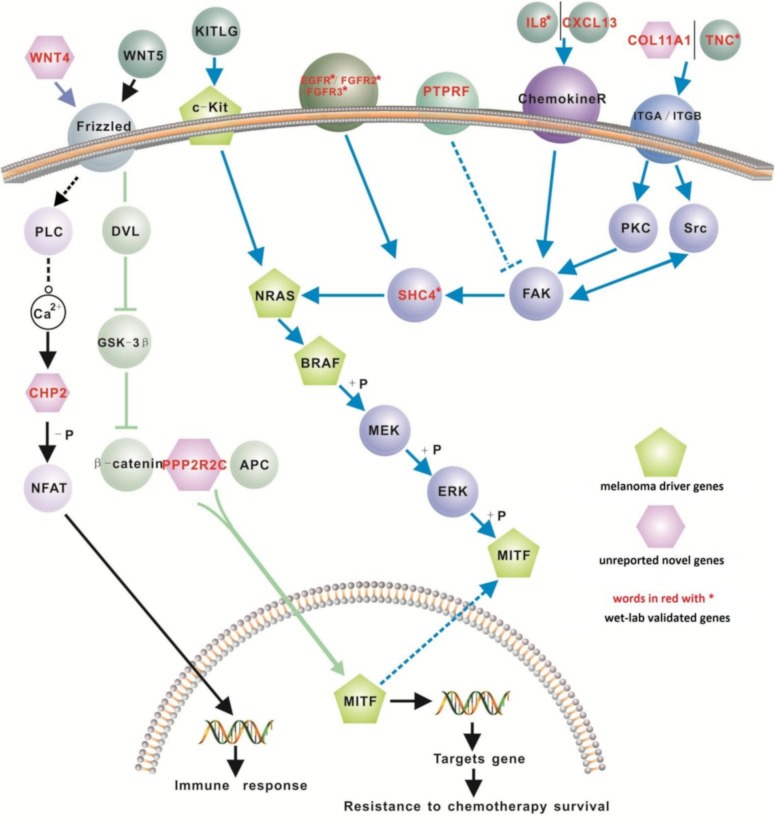
A new signaling network for melanoma. The signaling network is based on the complex interactions of the 12 signature genes (labeled in red) and the 4 melanoma driver genes (*BRAF, cKit, NRAS, MITF*) in 3 signaling pathways (MAPK, Ca^2+^ and WNT). Nine of these 12 genes (i.e., *EGFR, FGFR2, FGFR3, IL8, PTPRF, CXCL13, TNC, COL11A1, and SHC4*) closely interact with three driver genes (*NRAS, BRAF, and MITF*) in the MAPK signaling pathway: the remaining 3 genes include *WNT4, PPP2R2C and CHP2*, which also play important roles in WNT and Ca^2+^ signaling pathways.

We retrieved 31 pathways from the KEGG database where 12 genes in our proposed 200-gene signature were found to closely interact with the 4 melanoma driver genes in the MAPK, Ca^2+^ and WNT signaling pathways (Table 3). These 12 genes are *EGFR, FGFR2, FGFR3, IL8, PTPRF, TNC, CXCL13, COL11A1, CHP2, SHC4, PPP2R2C,* and *WNT4*. Based on these interactions we propose a new signaling network for melanoma (Fig. 2). Of these 12 genes, *CXCL13, SHC4, WNT4* and *CHP2* were detected only using our computational method (i.e., not reported before in melanoma microarray studies) but exhibit important positions in melanoma driver gene signaling pathways (Fig. 2). The biological pathways involving chemokine receptors, WNT, Ca^2+^ and MAPK signaling will have implications for melanomagenesis and metastatic progression.

**Table 3:** Pathways where the 12 genes closely interact with melanoma driver genes (BRAF, NRAS, cKIT and MITF).

No.	Pathways	Driver genes assocated pathway (BARF, NRAS, c-KIT, MITF)	IL8	FGFR3	PTPRF	TNC	SHC4	CXCL13	EGFR	WNT4	FGFR2	PPP2R2C	CHP2	COL11A1
1	hsa04010	MAPK signaling pathway		✓					✓		✓		✓	
2	hsa04012	ERBB signaling pathway					✓		✓					
3	hsa04060	cytokine-cytokine receptor interaction	✓					✓	✓					
4	hsa04062	chemokine signaling pathway	✓				✓	✓						
5	hsa04115	p53 signaling pathway												
6	hsa04144	endocytosis		✓					✓		✓			
7	hsa04360	axon guidance											✓	
8	hsa04370	VEGF signaling pathway											✓	
9	hsa04510	focal adhesion				✓	✓		✓					✓
10	hsa04530	tight junction										✓		
11	hsa04540	GAP junction							✓					
12	hsa04650	natural killer cell mediated cytotoxicity					✓						✓	
13	hsa04660	T cell receptor signaling pathway											✓	
14	hsa04662	B cell receptor signaling pathway											✓	
15	hsa04720	long-term potentiation											✓	
16	hsa04722	neurotrophine signaling pathway					✓							
17	hsa04810	regulation of actin cytoskeleton		✓					✓		✓			
18	hsa04910	insulin signaling pathway			✓		✓							
19	hsa04912	GnRH signaling pathway							✓					
20	hsa04916	melanogenesis								✓				
21	hsa05160	hepatitis C	✓						✓			✓		
22	hsa05166	HTLV-1 infection								✓				
23	hsa05200	pathways in cancer	✓	✓					✓	✓	✓			
24	hsa05212	pancreatic cancer							✓					
25	hsa05213	endometrial cancer							✓					
26	hsa05214	glioma					✓		✓					
27	hsa05215	prostate cancer							✓		✓			
28	hsa05218	melanoma							✓					
29	hsa05219	bladder cancer	✓	✓					✓					
30	hsa05220	chronic myeloid leukemia					✓							
31	hsa05223	non-small cell lung cancer							✓					

### Experimental validation of a MAPK pathway-associated subset in our 12-gene melanoma signature

**Figure 3:**
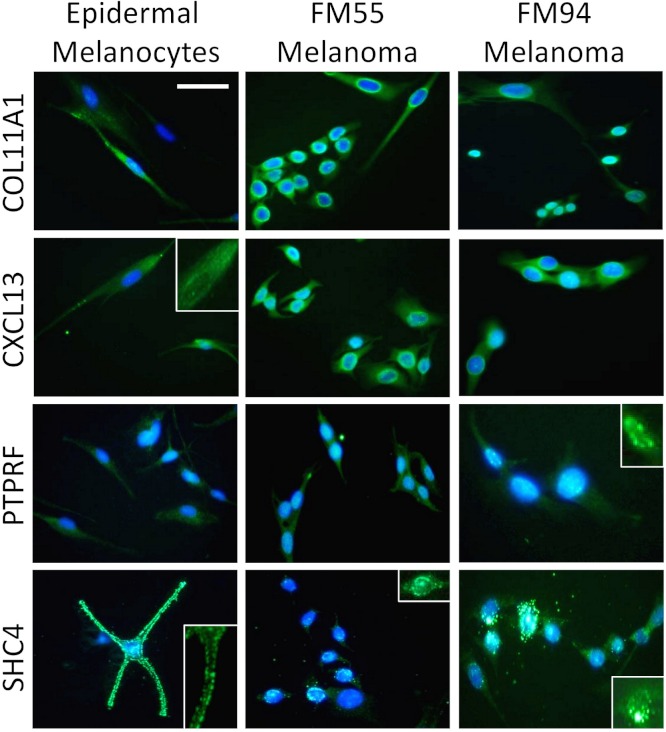
Immunocytochemical analysis of human melanocytes and melanoma cells *in vitro*. COL11A1, CXCL13, PTPRF and SHC4 proteins were upregulated (green fluorescence) in melanoma cells. Inserts show higher power views of expression, including when associated with the perinuclear region of the cell.

Four genes in our proposed 12-gene biomarker signature that appear in the MAPK signaling pathway (i.e., COL11A1, CXCL13, PTPRF, and SHC4) were selected for laboratory validation. Note that COL11A1, CXCL13, and PTPRF have not previously been reported to be associated with melanoma experimentally. COL11A1, CXCL13, PTPRF, and SHC4 were found to be over-expressed in two human melanoma cell lines (i.e., FM55 and FM94) compared to normal human epidermal melanocytes *in vitro* (Fig. 3). A significant degree of heterogeneity was observed in the expression pattern for these markers. For example, COL11A1, a secreted collagen protein, was observed at low levels in the cytoplasm of normal melanocytes, but more intensely in the perikayon of moderately-pigmented FM55 melanoma cells, and unexpectedly exhibited a nuclear/nuclear membrane association in the pigmented FM94 melanoma cells. Similarly, a weak cytoplasmic localization of CXCL13 in normal melanocytes appeared to shift towards the perikayon and nucleus of FM55 and FM94 melanoma cells respectively, as evidenced by co-localization with DAPI staining. Low level PTPRF expression in normal epidermal contrasted with higher expression (both cytoplasmic and nuclear) in melanoma cells. Finally, SHC4 expression was membranous in normal melanocytes contrasting with some punctuate nuclear membrane expression in melanoma cells (Fig. 3).

The expression of these four proteins was also assessed in normal human healthy skin and in melanoma patient tissue (both primary and metastatic melanoma). Using double immunofluorescence with a melanocyte lineage marker gp100, we assessed the relationship of the four test proteins with melanocytes or melanoma cells in these tumor biopsies. We included primary melanoma (in addition to metastatic melanoma) in our immunohistochemistry validation study because the expression levels for the 12 genes in our signature exhibited several fold level changes between primary melanoma and normal skin/benign nevi across 5 microarray datasets (Table S8).

COL11A1, CXCL13 and PTPRF were not detected in normal human epidermal melanocytes in situ (Fig. 4a). Some low level expression of SHC4 was detected in these normal pigment cells. By contrast, COL11A1 was expressed intensely by melanoma cells located in the dermis of both primary and metastatic melanoma (Fig. 4b). CXCL13 was strongly expressed in a minor subpopulation of tumor cells in primary melanoma, while a greater fraction of cells in metastatic melanoma tissue expressed this protein. By contrast, PTPRF was intensely expressed in the majority of tumor cells of both primary and metastatic melanoma cells. Finally SHC4 was found to be expressed in minor fraction of primary gp100-positive melanoma, but in most metastatic gp100-positive melanoma cells.

**Figure 4a:**
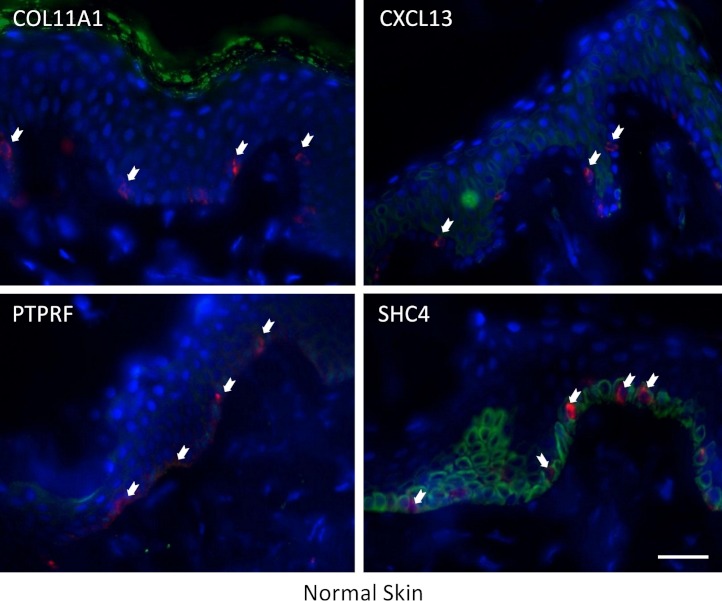
Immunohistochemical analaysis of COL11A1, CXCL13, PTPRF and SHC4 in normal human skin epidermis. Melanocytes were detected with an antibody (NKi/beteb) raised against the melanocyte-specific marker gp100 (red, arrows). COL11A1, CXCL13, PTPRF (shown in green) were not detected in normal epidermal melanocytes. SHC4 was expressed strongly in proliferating keratinocytes in the basal layer on the epidermis, and to some extent also in melanocytes (i.e. double positive cells in orange-yellow).

**Figure 4b:**
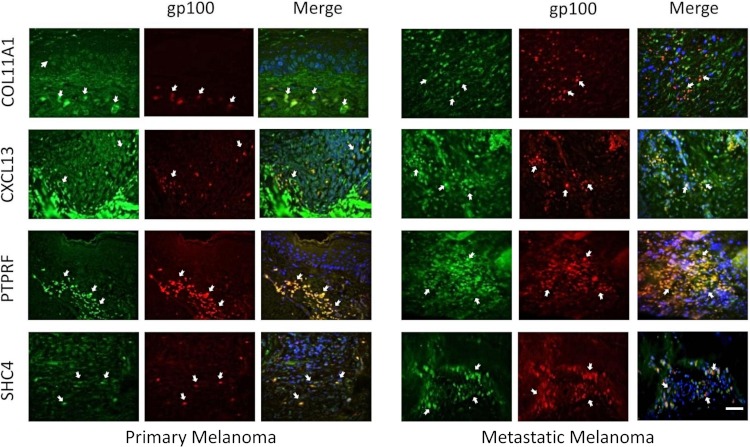
Immunohistochemical analaysis of COL11A1, CXCL13, PTPRF and SHC4 in primary and metastatic melanoma. Double staining of test protein (shown in green) and pigment cell lineage-specific marker gp100 (in red, arrows). Both immunoreactivites were merged with yellow/orange fluorescence indicating co-localization of these proteins in melanoma cells.

**Figure 5:**
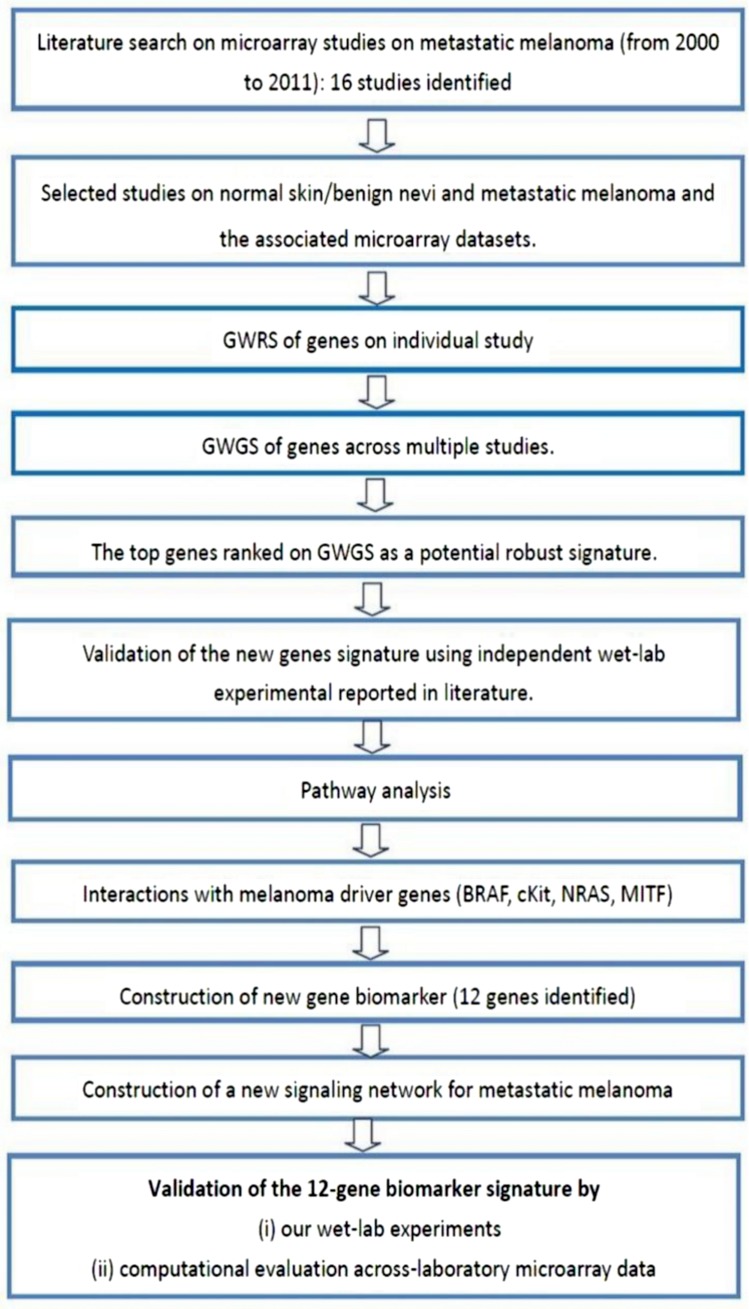
Experimental protocol of study.

### Computational evaluation of the robustness of a proposed 12-gene biomarker signature in distinguishing melanoma from normal skin and/or benign nevi

A computational evaluation of robustness of the proposed 12-gene signature, based on melanoma driver gene association, was performed for distinguishing melanoma from normal skin and/or benign nevi using cross-laboratory published data. This data evaluation is important to verify the robustness of a new biomarker for potential diagnostic application and/or possible therapeutic development. The support vector machine (so-called SVM model) classification model (Brown et al., 2000) and the ‘leave-one-out method’ are used to classify microarray datasets (Hoek et al., 2004; Smith, Hoek & Becker, 2005; Riker et al., 2008; Scatolini et al., 2010; Rose et al., 2011). Our results showed that these 12 genes achieved excellent classification accuracy ratios across these five datasets (i.e., average of 99.1%, Table 4). This result indicated that our 12-gene biomarker achieved a classification accuracy ratios that was identical or near identical to the classification accuracy ratios of the original individual studies. Importantly, the 12-gene biomarker signature achieved a much better performance on average than the signatures of Smith, Hoek & Becker (2005), Riker et al. (2008) and Scatolini et al. (2010), and very slightly less (0.44% less) classification accuracy than the signature of Hoek et al. (2004). It should be noted that the signature of Hoek et al. (2004) consisted of 589 genes, while our biomarker signature is very much shorter at just 12 genes.

**Table 4:** Classification accuracy of four original signatures on across-laboratory data.

Original signatures	GSE4570 (2004)	GSE4587 (2005)	GSE7553 (2008)	GSE12391 (2010)	GSE22301 (2011)	Average
(Hoek et al., 2004) (589)	100%	100%	97.78%	100%	100%	99.56%
(Smith, Hoek & Becker, 2005) (100)	71.43%	100%	97.78%	100%	100%	93.84%
(Riker et al., 2008) (65)	71.43%	100%	95.56%	100%	100%	93.40%
(Scatolini et al., 2010) (455)	85.71%	100%	97.78%	100%	100%	96.70%
New 12-gene biomarkers	100%	100%	95.56%	100%	100%	99.11%

## Discussion

There is poor congruence between gene signatures generated by different microarray-based melanoma studies (John et al., 2008; Bittner et al., 2000; Tímár, Gyorffy & Rásó, 2010). Unsurprisingly therefore, microarray-based melanoma gene biomarkers have had poor translation to clinical practice, and melanoma diagnosis is still based on clinical and histopathological features of the tumor (Schramm et al., 2011). To perform a meta-analysis on microarray gene expression data, Rhodes et al. (2002) introduced a model for combination of differentially-expressed genes based on their *p*-value in a statistical test. Here we propose a new and universally-applicable method to overcome some limitations of the Rhodes model (see Methods). Our new method measures firstly the ‘genome-wide relative significance’ (GWRS) as defined in an individual dataset followed by a ‘genome-wide global significance’ (GWGS) as defined as an assessment across multiple datasets. The robustness and effectiveness of our approach can be supported by several lines of evidence and validation.

First, a considerable number of novel genes (e.g., *GTAG1A/1B/2, GAGE1-8/12B-J, XAGE1A-E, IL8, IGF2/INS-IGF2, SHC4, LEP, TF, CYP3A5*, *TP63* and *GBP5*) revealed by our method were not identified as significant genes in the previous 16 melanoma microarray studies published between 2000 and 2011, but have still been confirmed as melanoma-associated by independent ‘wet-lab’ studies in the literature (Table S4).

Second, our method identified a core signature of 12 genes (*i.e., EGFR, FGFR2, FGFR3, IL8, PTPRF, TNC, CXCL13, COL11A1, SHC4, CHP2, PPP2R2C* and *WNT4*) that are closely associated with known melanoma driver genes. However, six signature genes (i.e., *IL8, SHC4, COL11A1, CHP2, PPP2R2C* and *WNT4*) were not reported previously by microarray-based melanoma studies, although two (i.e. *IL8* and *SHC4*) have been identified in independent wet-lab studies (Zhang et al., 2011; Fagiani et al., 2007; Pasini et al., 2009). This leaves *WNT4, CHP2, PPP2R2C* and *COL11A1* which have not been previously reported to be associated with melanoma. However, Fedida-Metula recently suggested a relationship between Ca^2+^ signaling members and *PP2A* and melanoma tumor growth (Fedida-Metula et al., 2012). CHP2 (‘calcineurin-like EF hand protein’) is involved in calcium signaling, while *PPP2R2C* is a member of the *PP2A* family.

Third, we validated the expression of MAPK-associated members (COL11A1, CXCL13, PTPRF, SHC4) of the 12-gene biomarkers in a comparative analysis of normal melanocytes and melanoma cells *in vitro* and in primary versus metastatic melanoma biopsy tissue *in situ*. All four markers were found to be preferentially associated with melanoma, being differentially expressed in primary and metastatic melanoma. Strikingly, COL11A1, CXCL13, and PTPRF were not detectable in epidermal melanocytes of normal healthy human skin epidermis. SHC4 was expressed at low levels in normal epidermal melanocytes, as previously shown (Fagiani et al., 2007).

The over-expression of COL11A1, CXCL13, PTPRF, and SHC4 in melanoma cells *in vitro* and *in situ* may reflect the observed over-expression of the associated genes in our microarray meta-analysis results. The considerably higher level of SHC4 expression in the perikaryon of melanoma cells is of note, and concurs with other studies showing restricted expression in melanomas, while only weakly expressed in normal melanocytes and benign nevi (Fagiani et al., 2007). There is evidence that SHC4 is highly expressed at the transition from radial growth phase to vertical growth phase and metastatic melanomas, contemporaneous with the acquisition of melanoma migratory competence and invasive potential (Fagiani et al., 2007; Pasini et al., 2009). This protein tyrosine phosphatase acts as a signaling molecule to regulate cell growth, differentiation, mitotic cycle, and oncogenic transformation (Junta et al., 2008). PTPRF usually is expressed in the cell membrane (i.e. is a receptor-type protein tyrosine phosphatase) where it interacts with β-catenin and like β-catenin may be translocated to the nucleus upon activation. The over-expression of COL11A1, CXCL13, PTPRF and SHC4 in our melanoma cell lines and primary and metastatic tissue, and their potential association with MAPK pathways suggests they could be specific biomarkers for melanoma and so potential therapeutic targets.

Our computational evaluation indicates that this new 12-gene biomarker signature achieves excellent diagnostic power in distinguishing metastatic melanoma from normal skin and benign nevus. The integrated analysis of these five microarray datasets has identified a robust 12-gene biomarker signature that includes six previously-unreported genes in melanoma. Further experimental validation of the role of these 12 signature genes in a revised signaling network may provide new insights into the underlying biological mechanisms driving the progression of melanoma. Moreover, given that the original signatures involved much larger numbers of genes (e.g., 589, 100, 65, 455 genes per signature), an excellent classification accuracy ratio performance was achieved by our melanoma biomarker signature with just 12 genes. This supports the view that our integrated approach extracts more informative genes than the original signatures, and from a clinical perspective our 12-gene signature could be a more valuable biomarker for melanoma in the clinical setting.

## Supplemental Information

10.7717/peerj.49/supp-1 Table S1 Selected 16 independent studies used in this study.

10.7717/peerj.49/supp-2 Table S2 Melanoma signatures of 16 original studies

10.7717/peerj.49/supp-3 Table S3 Metastatic melanoma signatures of 16 original studies

10.7717/peerj.49/supp-4 Table S4 The genes validated by independent wet-lab experimental studies.

10.7717/peerj.49/supp-5 Table S5 Publically available microarray datasets concerning metastatic melanoma

10.7717/peerj.49/supp-6 Table S6 Five microarray datasets for comparative study of normal skin/benign nevi and metastatic melanoma

10.7717/peerj.49/supp-7 Table S7 Novel 77 genes with original rank positions and sr values

10.7717/peerj.49/supp-8 Table S8 Fold change values of 12 signature genes between benign nevi/normal skin and primary melanoma.
